# Association analysis of rare variants near the *APOE* region with CSF and neuroimaging biomarkers of Alzheimer’s disease

**DOI:** 10.1186/s12920-017-0267-0

**Published:** 2017-05-24

**Authors:** Kwangsik Nho, Sungeun Kim, Emrin Horgusluoglu, Shannon L. Risacher, Li Shen, Dokyoon Kim, Seunggeun Lee, Tatiana Foroud, Leslie M. Shaw, John Q. Trojanowski, Paul S. Aisen, Ronald C. Petersen, Clifford R. Jack, Michael W. Weiner, Robert C. Green, Arthur W. Toga, Andrew J. Saykin, for the Alzheimer’s Disease Neuroimaging Initiative (ADNI)

**Affiliations:** 1 Center for Neuroimaging, Department of Radiology and Imaging Sciences, Indiana University School of Medicine, Indianapolis, IN USA; 2 Department of Medical and Molecular Genetics, Indiana University School of Medicine, Indianapolis, IN USA; 3 Center for Computational Biology and Bioinformatics, Indiana University School of Medicine, Indianapolis, IN USA; 4 Department of Pathology and Laboratory Medicine, University of Pennsylvania School of Medicine, Philadelphia, PA USA; 5 Department of Neuroscience, University of California-San Diego, San Diego, CA USA; 6 Department of Neurology, Mayo Clinic Minnesota, Rochester, MN USA; 7 Department of Radiology, Mayo Clinic Minnesota, Rochester, MN USA; 8 Departments of Radiology, Medicine, and Psychiatry, University of California-San Francisco, San Francisco, CA USA; 9 Department of Veterans Affairs Medical Center, San Francisco, CA USA; 10 Division of Genetics, Department of Medicine, Brigham and Women’s Hospital and Harvard Medical School, Boston, MA USA; 11 The Institute for Neuroimaging and Informatics and Laboratory of Neuro Imaging, Keck School of Medicine of USC, University of Southern California, Los Angeles, CA USA; 12 Indiana Alzheimer’s Disease Center, Indiana University School of Medicine, Indianapolis, IN USA; 13 Department of Biomedical and Translational Informatics, Geisinger Health System, Danville, PA USA; 14 Department of Biostatistics, School of Public Health, University of Michigan, Ann Arbor, MI USA; 15 Department of Electrical and Computer Engineering, State University of New York at Oswego, Oswego, NY USA

**Keywords:** Whole genome sequencing, Rare variants, Near *APOE*, ADNI, CSF, Neuroimaging

## Abstract

**Background:**

The *APOE* ε4 allele is the most significant common genetic risk factor for late-onset Alzheimer’s disease (LOAD). The region surrounding *APOE* on chromosome 19 has also shown consistent association with LOAD. However, no common variants in the region remain significant after adjusting for *APOE* genotype. We report a rare variant association analysis of genes in the vicinity of *APOE* with cerebrospinal fluid (CSF) and neuroimaging biomarkers of LOAD.

**Methods:**

Whole genome sequencing (WGS) was performed on 817 blood DNA samples from the Alzheimer’s Disease Neuroimaging Initiative (ADNI). Sequence data from 757 non-Hispanic Caucasian participants was used in the present analysis. We extracted all rare variants (MAF (minor allele frequency) < 0.05) within a 312 kb window in *APOE’*s vicinity encompassing 12 genes. We assessed CSF and neuroimaging (MRI and PET) biomarkers as LOAD-related quantitative endophenotypes. Gene-based analyses of rare variants were performed using the optimal Sequence Kernel Association Test (SKAT-O).

**Results:**

A total of 3,334 rare variants (MAF < 0.05) were found within the *APOE* region. Among them, 72 rare non-synonymous variants were observed. Eight genes spanning the *APOE* region were significantly associated with CSF Aβ_1-42_ (*p* < 1.0 × 10^−3^). After controlling for *APOE* genotype and adjusting for multiple comparisons, 4 genes (*CBLC*, *BCAM*, *APOE, and RELB*) remained significant. Whole-brain surface-based analysis identified highly significant clusters associated with rare variants of *CBLC* in the temporal lobe region including the entorhinal cortex, as well as frontal lobe regions. Whole-brain voxel-wise analysis of amyloid PET identified significant clusters in the bilateral frontal and parietal lobes showing associations of rare variants of *RELB* with cortical amyloid burden.

**Conclusions:**

Rare variants within genes spanning the *APOE* region are significantly associated with LOAD-related CSF Aβ_1-42_ and neuroimaging biomarkers after adjusting for *APOE* genotype. These findings warrant further investigation and illustrate the role of next generation sequencing and quantitative endophenotypes in assessing rare variants which may help explain missing heritability in AD and other complex diseases.

## Background

The number of individuals with late-onset Alzheimer’s disease (LOAD) is rapidly increasing and predicted to triple by 2050 with the increasing population of aging adults [1]. The heritability of LOAD was predicted to be up to 80% based on twin studies [2] and large-scale genome-wide association studies (GWAS) have recently led to the identification and confirmation of approximately 22 LOAD-associated genes including *APOE* (Apolipoprotein E), the best established and most significant susceptibility gene for LOAD [3]. The association of *APOE* with LOAD has been replicated and validated in many studies from different populations [4]. The *APOE* ε4 allele increases an individual’s risk for developing LOAD and also reduces age-at-onset in patients with LOAD in a dose-dependent manner, while the *APOE* ε2 allele appears to reduce the risk for LOAD [5]. Furthermore, GWAS studies have repeatedly identified several susceptibility loci for LOAD near the 19q13 on the chromosome 19 including *APOE* and *TOMM40* (translocase of outer mitochondrial membrane 40 homolog) [3, 6]. In particular, *TOMM40* has the second most significant SNP (single nucleotide polymorphism) associated with LOAD and multiple LOAD-related neuroimaging phenotypes in the 19q13 region [7–9]. However, conditional analyses strongly suggested that this effect is due to *APOE* [10, 11]. As *APOE* and *TOMM40* are in strong linkage disequilibrium (LD), it is not easy to attribute an *APOE*-independent role of *TOMM40* in the risk of LOAD development, although *TOMM40* is essential for protein trafficking into mitochondria and mitochondrial dysfunction has been widely implicated in LOAD pathophysiology. Several groups investigated the association between a variable length poly-T polymorphism (poly-T) at rs10524523 within *TOMM40* and LOAD, and yielded contrasting results [12–16]. Recently, Jun et al. comprehensively evaluated the association of risk and age at onset of LOAD with common SNPs (MAF (minor allele frequency) > 5%) and poly-T repeat in the *APOE* region using approximately 23,000 cases and controls, and found no significant independent association after adjusting for *APOE* genotype [16]. Highly significant results, after adjusting for *APOE* genotype, are unlikely in view of the very strong LD in this region.

Up to 50% of LOAD heritability remain unexplained by all of the known LOAD susceptibility genes including *APOE* and a substantial missing heritability for LOAD remains to be identified [17]. The advent of high throughput next generation sequencing such as whole genome sequencing (WGS) to identify variation in human genes has created unprecedented opportunities to discover genetic factors that influence disease risk in the field of human genetics [18, 19]. Several recent reports show that deep re-sequencing of GWAS-implicated loci and WGS-based association studies can identify independent functional rare variants with large effects on diseases including LOAD pathogenesis [20–22].

Two neuropathological hallmarks of the AD brain are extracellular amyloid-β plaques and intracellular neurofibrillary tangles. Studies have shown decreased concentrations of the CSF Aβ_1–42_ peptide and increased concentrations of total tau (t-tau) and hyperphosphorylated tau (p-tau) in AD compared with cognitively normal elders [23, 24]. Here we performed a gene-based association analysis of rare variants within genes in the vicinity of *APOE* with cerebrospinal fluid (CSF) and LOAD-related neuroimaging markers using a WGS data set (*N* = 757) from the Alzheimer’s Disease Neuroimaging Initiative (ADNI) cohort. Our results strongly suggest rare variants in the region surrounding *APOE* on chromosome 19 were significantly associated with LOAD-related CSF Aβ_1-42_ and neuroimaging biomarkers.

## Methods

### Study participants

All individuals included in this study were participants of the longitudinal Alzheimer’s Disease Neuroimaging Initiative (ADNI) initiated in 2004, especially its subsequent extensions (ADNI-GO/2). Information about ADNI has been published previously and can be found at http://www.adni-info.org [25, 26]. All data were downloaded from the ADNI data repository (http://www.loni.usc.edu/ADNI/). All participants provided written informed consent at the time of enrollment for imaging and genetic sample collection and study protocols were approved by each participating sites’ Institutional Review Board (IRB).

For the control for population substructure, we restricted our analyses to participants with non-Hispanic Caucasian ancestry determined by using HapMap 3 genotype data and the multidimensional scaling (MDS) analysis (http://www.hapmap.org) [18, 19, 27]. Participants aged 55–90 to be used in this analysis include 259 cognitively normal older individuals (CN), 219 individuals with early mild cognitive impairment (MCI), 232 individuals diagnosed with late MCI, and 47 individuals diagnosed with AD.

### Whole genome sequencing (WGS) analysis

WGS data from 817 ADNI participants were downloaded from the ADNI data repository (http://www.loni.usc.edu/ADNI/). An established next generation sequencing analysis pipeline based on GATK previously described was used to process ADNI WGS data performed on blood-derived genomic DNA samples and sequenced on the Illumina HiSeq2000 using paired-end read chemistry and read lengths of 100 bp at 30-40X coverage (http://www.illumina.com) [28]. We extracted all variants (SNPs and short indels) within a 312 kb region in *APOE*’s vicinity including 12 genes.

### Neuroimaging analysis

T1-weighted brain MRI scans were processed using previously described automated MRI analysis techniques [29], whole-brain voxel-based morphometry (VBM) and FreeSurfer software [30, 31]. [^18^F]Florbetapir PET scans were pre-processed as described [30] and intensity normalized by the whole cerebellum. These normalizations yielded standardized uptake value ratio (SUVR) images [32].

### Statistical analysis

The SKAT-O software was used to perform a gene-based association analysis of all WGS-identified rare SNPs and short indels (MAF < 0.05) in the *APOE* cluster region [33]. We performed an association analysis first using only all SNPs and second using all SNPs plus short indels. Baseline CSF measurements (Amyloid-β 1–42 peptide (Aβ_1-42_), total tau (t-tau), and tau phosphorylated at the threonine 181 (p-tau_181p_)) were downloaded [34]. GWAS of CSF biomarkers found that several SNPs in *TOMM40* and *APOE* are significantly associated with Aβ_1-42_ [34]. Thus, for the CSF analysis, we used CSF Aβ_1-42_ as a quantitative phenotype and age, gender, and *APOE* genotype as covariates. For the neuroimaging analysis, age, gender, year of education, MRI field strength, total intracranial volume (ICV), and *APOE* genotype were as covariates. We considered associations with *p* < 0.0042 (=0.05/12) to be significant in order to control for multiple comparisons.

## Results

### Sequencing of chromosome 19q13 region

Within a 312 kb window in *APOE*’s vicinity spanning 12 genes, we found 683 common variants (618 SNPs and 65 indels) and 3,334 rare variants (3,040 SNPs and 294 indels) (Table 1). Among 4,017 variants, there are 147 exonic and 2,159 intronic variants. Of 147 exonic variants, we found 1 frameshift and 3 nonframeshift indels, 72 nonsynonymous and 51 synonymous SNPs, and 20 unknown variants.

**Table 1:** Number of common and rare variants (SNPs and Indels) of 12 genes near the *APOE* region

Gene	Common variant (MAF ≥ 5%)		Rare variant (MAF < 5%)	
	SNP	Indel	SNP	Indel
*BCL3*	67	9	408	42
*CBLC*	28	5	361	52
*BCAM*	41	2	327	25
*PVRL2*	190	29	513	65
*TOMM40*	32	2	154	11
*APOE*	13	1	51	3
*APOC1*	23	2	102	7
*APOC1P1*	21	3	113	9
*APOC4*	19	4	90	13
*APOC2*	27	2	61	3
*CLPTM1*	105	7	456	34
*RELB*	52	7	404	30
Total	618	65	3,040	294

### Association of rare variants near the *APOE* region with CSF Aβ_1-42_

Gene-based association analysis of rare SNPs near the *APOE* region identified three genes (*TOMM40*, *APOE*, and *APOC1*) that achieved a genome-wide significant association with CSF Aβ_1-42_ (*p* < 5 × 10^−7^) (Table 2) and the most significant association was between *APOC1* and CSF Aβ_1-42_. After controlling for *APOE* genotype and adjusting for multiple comparisons based on a Bonferroni threshold (*p* < 0.05/12 = 0.0042), 4 genes (*CBLC*, *BCAM*, *APOE*, and *RELB*) remain significant. The strongest significant association was observed at the *BCAM* gene (*p* = 0.0006). There were about 10% short indels of all rare variants near the *APOE* region. The results of gene-based association of both rare SNPs and short indels near the *APOE* region with CSF Aβ_1-42_ were almost same as the association results of only rare SNPs (Table 2).

**Table 2:** Gene-based association results (*p*-values) of rare variants (MAF < 5%; SNPs and Indels) of genes near the *APOE* region with CSF biomarker Aβ_1-42_ with and without adjusting for *APOE* genotypes

Gene	SNP + Indel		SNP	
	*p*-value	*p*-value adjusted for *APOE* genotype	*p*-value	*p*-value adjusted for *APOE* genotype
*BCL3*	8.38E-04	0.0056	7.60E-04	0.0054
*CBLC*	7.07E-05	0.0011	7.35E-05	0.0013
*BCAM*	1.97E-04	0.0005	2.49E-04	0.0006
*PVRL2*	3.55E-05	0.3842	4.40E-05	0.4605
*TOMM40*	6.84E-07	0.0922	5.01E-07	0.0880
*APOE*	3.35E-10	0.0039	4.08E-07	0.0036
*APOC1*	6.18E-11	0.2394	2.85E-11	0.1636
*APOC1P1*	4.43E-02	0.0145	6.16E-02	0.0097
*APOC4*	2.11E-02	0.2062	1.60E-02	0.1642
*APOC2*	2.23E-01	0.5363	1.64E-01	0.5102
*CLPTM1*	1.02E-02	0.0438	9.12E-03	0.0377
*RELB*	2.36E-04	0.0053	1.51E-04	0.0042

### Association of rare variants near the *APOE* region with neuroimaging (MRI, PET)

**Fig. 1:**
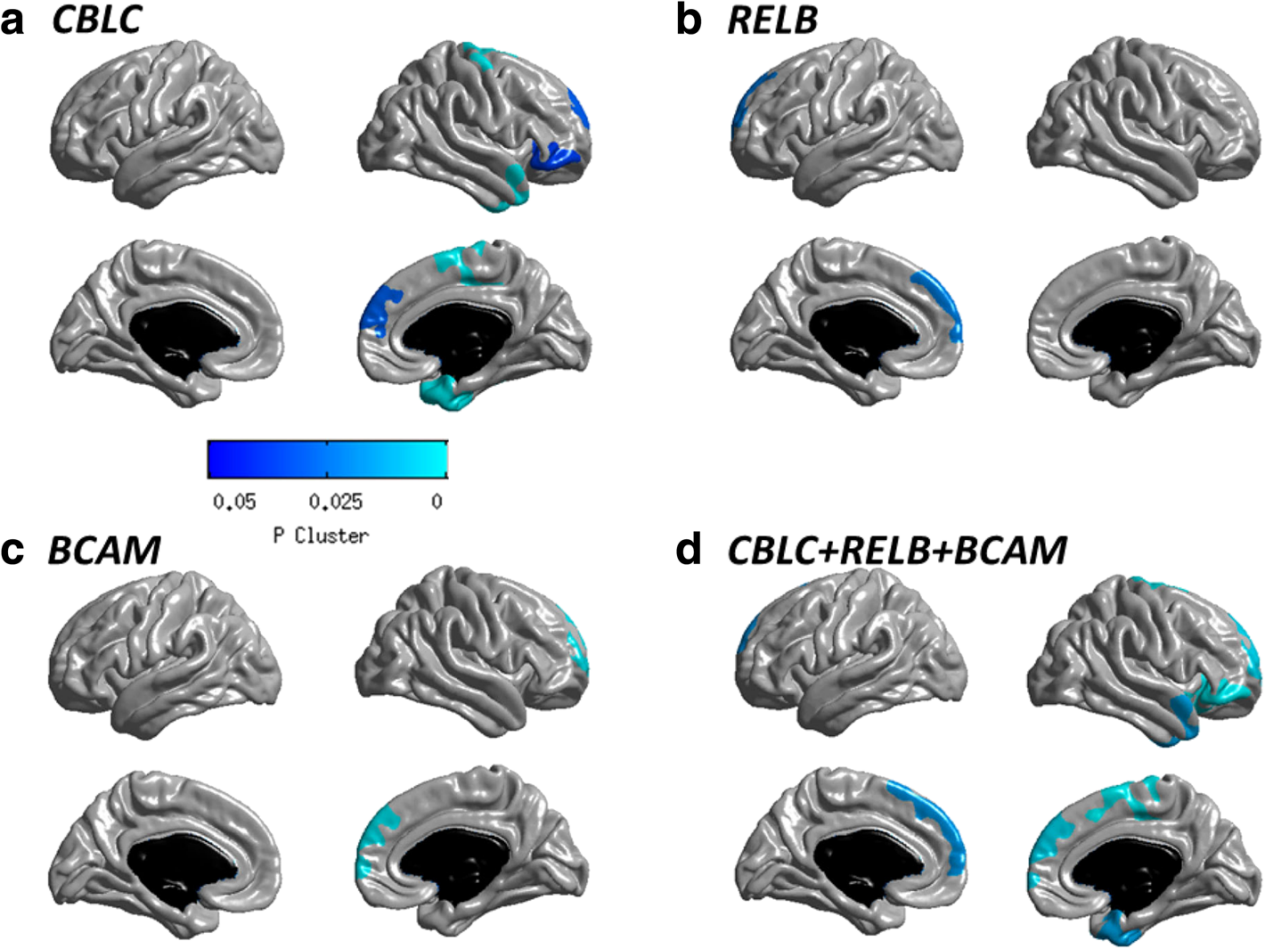
Surface-based whole-brain analysis results. A whole-brain multivariate analysis of cortical thickness was performed on a vertex-by-vertex basis to visualize the topography of genetic association in an unbiased manner. Statistical maps were thresholded using a random field theory adjustment to a corrected significance level of *p* = 0.05. **a** *CBLC*. **b** *RELB.* **c** *BCAM*. **d** *CBLC + RELB + BCAM*

**Fig. 2:**
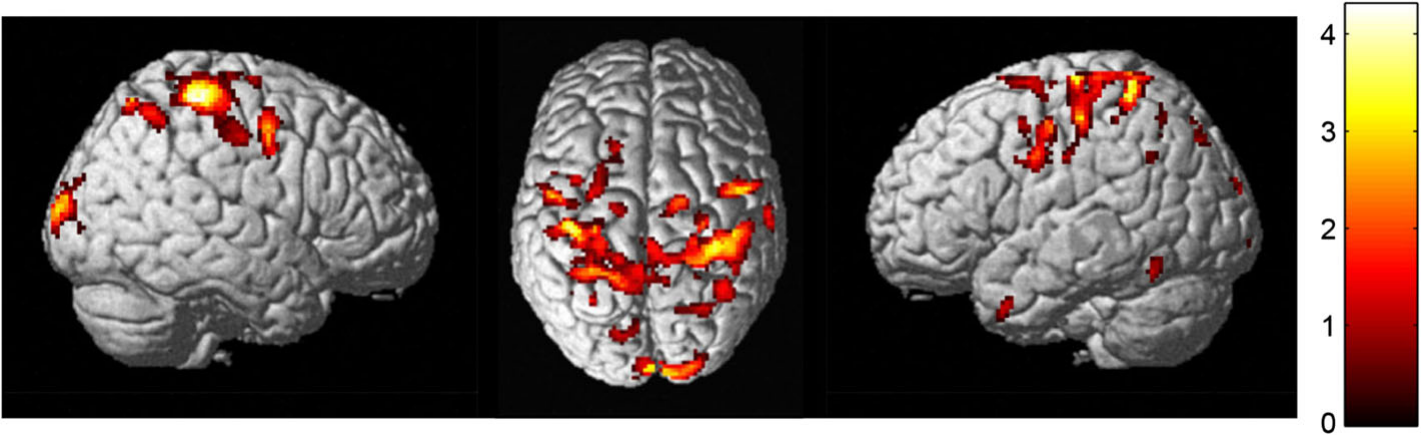
Voxel-wise analysis results of [^18^F]Florbetapir positron emission tomography (PET). A whole-brain analysis of cerebral amyloid deposition was performed on a voxel-by-voxel basis to visualize the topography of genetic association (*RELB*) in an unbiased manner. Figure is displayed at an uncorrected *p* value <0.005 and minimum voxel size (k) = 27 voxels

To examine the LOAD-related neuroimaging biomarker association of all rare variants in 3 genes (*CBLC*, *BCAM*, and *RELB*) significantly associated with CSF Aβ_1-42_ after adjusting for *APOE* genotype, a detailed whole-brain multivariate analysis of cortical thickness (MRI) and amyloid-β burden ([^18^F]-florbetapir PET) was performed to detect brain regions of associations of a single polygenic risk score. A single polygenic risk score was determined by collapsing all rare variants and counting minor alleles with a dominant genetic model. Figure 1 displays the results of the main effect of all rare variants after adjusting for *APOE* genotype in a surface-based cortical thickness whole brain analysis. Highly significant clusters associated with the risk score were found in temporal lobes including the entorhinal cortex, where AD pathology primarily begins, frontal lobe regions for *CBLC*, and temporal lobe regions for *BCAM* and *RELB*, where subjects having high risk scores showed thinner mean cortical thickness compared with the participants having lower risk scores. A polygenic risk score of all rare variants in 3 genes (*CBLC*, *BCAM*, *RELB*) was associated with multifocal brain atrophy, predominantly in the temporal and bilateral frontal lobes (Fig. 1d). Fig. 2 shows the association of all rare variants in *RELB* with cortical amyloid burden from voxel-wise analysis of the effect of rare variants on amyloid accumulation measured by [^18^F]-florbetapir PET at a voxel-wise threshold of *p* < 0.005 (uncorrected). The color scale indicates regions where the risk scores were associated with higher amyloid burden after adjusting for *APOE* genotype. The significant clusters were observed in the bilateral frontal and parietal lobes.

### Association of common SNPs near the *APOE* region with CSF Aβ_1-42_

The association analysis of common SNPs near the *APOE* region was performed using PLINK set-based tests and permutation while considering the linkage disequilibrium structure of SNPs and identified one significant gene (*BCL3*) passed a Bonferroni threshold after adjusting for *APOE* genotype (*p* = 0.0005; Table 3). The association results remain almost unchanged when both common SNPs and short indels were used.

**Table 3:** Gene-based association results (*p*-values) of common variants (MAF ≥ 5%; SNPs and Indels) of genes near the *APOE* region with CSF biomarker Aβ_1-42_ with and without adjusting for *APOE* genotypes

Gene	SNP + Indel		SNP	
	*p*-value	*p*-value adjusted for *APOE* genotype	*p*-value	*p*-value adjusted for *APOE* genotype
*BCL3*	0.0013	0.0005	0.0019	0.0005
*CBLC*	0.0006	0.0122	0.0005	0.0128
*BCAM*	0.0014	0.0132	0.0016	0.0131
*PVRL2*	<1.0E-05	0.6852	<1.0E-05	0.6665
*TOMM40*	<1.0E-05	1.0000	<1.0E-05	1.0000
*APOE*	<1.0E-05	0.1380	<1.0E-05	1.0000
*APOC1*	<1.0E-05	1.0000	<1.0E-05	1.0000
*APOC1P1*	<1.0R-05	1.000	<1.0E-05	1.0000
*APOC4*	0.1718	1.000	0.1437	1.0000
*APOC2*	0.0406	0.0621	0.0152	0.0570
*CLPTM1*	0.0198	0.0467	0.0331	0.0464
*RELB*	0.0515	0.6551	0.2485	0.6095

## Discussion and Conclusions

We show for the first time to our knowledge that rare variants within genes near the *APOE* region are significantly associated with a LOAD biomarker CSF Aβ_1-42_ after adjusting for *APOE* genotype. Our results indicated that four genes (*CBLC*, *BCAM*, *APOE,* and *RELB*) remained significant after correcting for multiple comparisons. In addition, gene-based association analysis of common variants identified one significant gene *BCL3*. Whole-brain surface-based analysis identified highly significant clusters associated with rare variants of *CBLC* in temporal lobe regions including the entorhinal cortex and frontal lobe regions.

*BCL3* (B-cell CLL/lymphoma 3) gene functions as a transcriptional co-activator involved in cell replication and apoptosis that activates through its association with NF-κB homodimers [35]. *BCL3* gene is associated with genetic linkage with late-onset Familial Alzheimer’s disease as well as chronic lymphocytic leukemia [36–38]. *RELB* (RELB proto-oncogene, NF-κB subunit) gene is a member of NF-κB family of transcriptional factors. Among its related pathways are immune system and interleukin-3, 5 and GM-CSF signaling. NF-κB plays a central role in the inflammatory and immune responses and controls cell proliferation and protects the cell from apoptosis [39]. NF-κB is a major transcription factor and activated in AD patients. Amyloid beta accumulation is a potential activator of NF-κB in primary neurons [40]. *CBLC* (Cbl proto-oncogene C, E3 ubiquitin protein ligase) gene is the member of the Cbl family of E3 ubiquitin ligases. Cbl proteins play an important role in cell signaling through the ubiquitination and subsequent downregulation of the tyrosine kinases. *BCAM* (basal cell adhesion molecule) gene encodes a glycoprotein expressed on cell surfaces [41]. *BCAM* is a member of the immunoglobulin superfamily and a receptor for the extracellular matrix protein, laminin α-5. *BCAM* may play a role in intracellular signaling. *BCAM* is related to the Lutheran glycoprotein, which is a specific marker of brain capillary endothelium, which forms the blood brain barrier (BBB) in vivo [42, 43].

ADNI is a unique cohort and the only large WGS data set of LOAD with CSF Aβ_1-42_ and neuroimaging data also available. However, a limitation of the present report is that we used a modest sample size (*n* = 757) of whole genome sequencing data for genetic analysis. Therefore, validation in independent and larger cohorts is warranted.

In conclusion, we used whole genome sequencing to perform an association analysis of rare variants within genes near the *APOE* region with CSF Aβ_1-42_ and neuroimaging biomarkers of LOAD. Importantly, our results implicate this region or these genes contain additional explanatory information with regard to LOAD endophenotypes above and beyond that conferred by *APOE* genotype. Overall, combining whole genome sequencing and LOAD-related quantitative endophenotypes adds to the growing understanding of the genetics of LOAD and holds promise for discovery of rare variants involved in neurodegeneration and other brain disorders, further nominating novel potential diagnostic and therapeutic targets.
